# Investigation of Childhood Traumas of Individuals Who Experienced Parental Death in Childhood or Adolescence in Turkey

**DOI:** 10.1007/s40653-024-00629-2

**Published:** 2024-03-15

**Authors:** Serap Daşbaş, Rasim Babahanoğlu, Nur Feyzal Kesen, Semra Saruç, Meliha Funda Afyonoğlu

**Affiliations:** 1 Faculty of Health Sciences, Department of Social Work, Selçuk University, Alaaddin Keykubat Campus, Konya, Selcuklu Turkey; 2 Faculty of Health Sciences, Department of Social Work, Hitit University, Çorum Merkez/Çorum, Turkey; 3 Faculty of Health Sciences, Department of Social Work, Anadolu University, Eskişehir, Turkey

**Keywords:** Loss of parent, Childhood trauma, Family, Trauma-informed social work practice, School social work

## Abstract

This study investigates the childhood traumas of individuals who experienced parental death in childhood or adolescence in Turkey. Participants (*n* = 382; 63.1% female and 36.9% male; M_AGE_=29.41, SD = 9.6) are composed of individuals who lost either or both of their parents before the age of 18. The findings indicated that there is a significant difference between the subdimensions of Childhood Trauma Questionaire and the study variables including gender, marital status, educational status, number of siblings, gender of the deceased parent, the caregiver after the parental death, relationship with the surviving parent and the age at the time of parental death (*p* < .05). The results further show that individuals who lost their parents before the age of 10, who lost their mothers, and who received care from other relatives instead of parents are exposed to more neglect and abuse. By identifying childhood traumas in individuals and examining related variables, the findings contribute trauma-informed social work practices. Based on the results, it is recommended that school social work programs focus on monitoring the relationship between caregivers and children, as well as enhancing the psychosocial well-being of students. These programs can also serve as a means to raise awareness among professionals and students.

**Supplementary Information:**

The online version contains supplementary material available at 10.1007/s40653-024-00629-2.

## Introduction

Since the times of Freud, psychologist, psychiatrics and academics have been discussing the effects of childhood experiences in adult’s life. Negative emotions elicited by various problems in childhood such as parental neglect or abuse, the witnessing of violence between parents, coming from a fragmented family, parental death or various parental problems considerably influence the child’s life styles, relationships and personality (Bulut, 1996). These adverse experiences can significantly impact a child’s preparedness to face the future and their coping abilities and interpersonal relationships (Turkish Association of Psychologists, 2014). When these negative emotions threaten or disrupt an individual’s core well-being, they can give rise to traumatic events, and childhood traumas are well-known for their long-lasting effects that persist into adulthood. Among these childhood traumas, actions or inactions by parents or caregivers that result in physical, mental, sexual, or social harm to the child can be considered as some of the most impactful traumatic experiences (Taner & Gökler, 2004; Howell et al., 2016).

Parental death is seen as one of the most traumatic experiences in childhood (Gertler et al., 2004; Haine et al., 2006) and various studies have shown that parental death or separation affects psychopathology in adulthood and causes many problems involving anxiety, and depression (Finkelstein, 1988; Dowdney, 2000; Cerel et al., 2006; Tyrka et al., 2008; Örsel et al., 2011). In terms of learned helplessness and coping mechanisms, the consequences of parental death, including lack of a parent model and social support, may place further strain on the child and trigger depression in adulthood (Takeuchi et al., 2003). It was also reported that parental death might give rise to negative outcomes such as mental health problems, low academic performance, low self-respect, and higher external locus of control (Dowdney, 2000; Zoroğlu et al., 2001). There is also a growing body of the literature discussing the relationship between parental death and risk of suicide (Feigelman et al., 2017; Burrell et al., 2018; Hua et al., 2019).

This paper aims to investigate childhood traumas of individuals who have experienced parental death before the age of 18 and to evaluate whether there is a relationship between childhood trauma and certain variables related to the time of parental death and the current situation of the individual. Determining the childhood traumas of individuals as well as variables related to these traumas like gender, age, gender of the deceased parent, the caregiver after the death of the parent, and his/her relationship with the surviving parent are thought to make important contributions to the social work practice and literature.

Although there are studies on childhood traumas of children who are placed under protection in Turkey (Kesen et al., 2016) and of adults studying at university or utilizing clinical services (Bostancı et al., 2006; Uluğ, 2008), there is no study in Turkey which takes this specific sample as a research group. In other words, rather than taking parental death as a variable, our study treats this group as a sample. Therefore, we can say that although parental death is considered as a variable in research, no social work study in Turkey takes them as a sample group unless they have access to clinical or social work systems. Thus, we believe that we will fill an important gap in the literature.

In the international literature, while there is a wealth of research on childhood trauma globally, studies focusing on this particular non-clinical sample group from the perspective of social work are quite limited (Spratt et al., 2019). The same result is obtained when the research using the Childhood Trauma Questionaire (CTQ) as a research tool are analyzed. Internationally, the CTQ has been used in various clinical and non-clinical studies in the literature. In clinical studies, the CTQ was used for individuals with eating disorders, outpatient psychotherapy patients, inpatient psychiatry patients, people with body dysmorphic disorder, and individuals with substance use disorder; whereas, in non-clinical studies, it was used for college students, women who participate in welfare programs, foster-parents, parents of children with mental disabilities, child welfare workers, veteran soldiers, and victims of interpersonal violence (Baker & Maiorino, 2010). Similarly, numerous clinical and non-clinical studies used the CTQ with various samples in Turkey (Yüksel & Çifçi, 2017; Erol et al., 2013; Demirkapı-Şahin, 2013; Örsel et al., 2011; Özen et al., 2007; Evren & Ögel, 2003), and specifically the relationship between childhood trauma and depression was studied by various researchers (Bostancı et al., 2006; Uluğ, 2008; Erol et al., 2013). We believe that the inclusion of a social work perspective into this clinical, psychological and psychiatric research literature with this specific sample will make an important contribution to the trauma and social work literature.

Another result inferred from the literature is that different studies reached different results about the relationship between childhood trauma and related variables such as gender, age and surviving parents, and the literature highlights inconclusiveness of these results. We believe that these differences arose due to social, cultural, economical and political systems of the countries as well as families and methodological issues. Therefore, although we do not claim that we reached a conclusive result in the trauma literature, we believe that the inclusion of this sample will contribute to the social work literature both locally and internationally.

Finally, we can say that our sample is a possible client group for trauma-informed social work practice that can be defined as understanding the present situation of the client without assuming that the client is a survivor of trauma and without centering the intervention strategies on past trauma (Knight, 2015). As the trauma-informed social work literature suggests, social workers should be aware of the significant impact of childhood trauma on adults’ current issues. These current problems should be understood within the context of past experiences without resorting to reductionism (Levenson, 2017). Consequently, considering that our sample consisted of individuals who did not receive any psychological or social work support following the loss of a parent, we believe that our research will shed light on their hidden traumas and contribute to the adoption of trauma-informed practices among social workers. In other words, we believe that our sample needed social work intervention and support in the times of parental death, yet they could not utilize social services, their traumas remained invisible, and they may become potential clients of social workers today. For example, it is possible for social workers to face them in divorce, family counselling or in social aid services. Therefore, we believe that determining the factors that differentiate childhood traumas will enhance the knowledge about invisible adults who may bear the consequences of childhood trauma. As a result, we think that our research will contribute to the trauma-informed literature and the practice of social work.

In light of these, this research aims to evaluate childhood traumas of individuals who experienced parental death in childhood or adolescence and its relation to different variables (gender age, marital status, educational status, family income, number of siblings, order of siblings, the deceased parent, the caregiver after the death of the parent, the relationship with the surviving parent, participant’s age at the time of parental death).

## Method

The study has a quantitative design and utilized relational-screening model. Utilizing the snowball sampling technique, 382 participants from different cities of Turkey, who lost their parent(s) in childhood or adolescence and who were not placed under protection were included in the study.

### Data Collection Procedures

The sample was reached by using the snowball sampling technique. We recruited 40 social work students from a university. The students were selected according to their grades in the Research Techniques and Children Under Protection courses offered by the Department of Social Work. The students were informed about both the Personal Information Form and the CTQ and were asked to find up to 10 individuals who experienced parental loss in childhood and adolescence and had no experience with social work and psychological systems during and after experiencing parental death.

### Participants

Among 382 participants, 63.1% were female and 36.9% were male. The mean age of participants was 29.41 (SD: 9.60). While 39.1% of the participants were primary school graduates, 40.7% of the participants were high-school graduates. 50% of the participants were married and 56.4% of the participants had four or more siblings. 34.5% lost their parents at ages between one and ten. While 31.9% of the participants lost their mother, 63.6% of the participants lost their fathers. The sociodemographic characteristics of the participants are presented in Table 1.

**Table 1:** Sociodemographic characteristics of the participants

Sociodemographic characteristics	Number	(%)
**Gender**		
Female	241	63.1
Male	141	36.9
**Age Group**		
18–23	138	36.1
24–29	59	15.4
29–35	61	16.0
36–53	124	32.5
**Educational Status**		
Primary School	149	39.1
High School	155	40.7
College	77	20.2
**Marital Status**		
Single	191	50
Married	191	50
**Family Income**		
0-500	125	32.7
501-1,100	78	20.4
1,101-2,100	102	26.7
2,101 and above	77	20.1
**Number of siblings**		
1–3	163	43
4–6	165	43.5
7 and above	51	13.5
**Age at the time of parental death**		
1–10	132	34.5
11–18	241	63.0
**Deceased parent**		
Mother	122	31.9
Father	243	63.6
Mother and Father	17	4.4
**The parent’s age at death**		
19–35	102	26.7
36–45	162	42.4
46–62	118	30.8
**Caregiver after the parental death**		
The other parent	314	82.1
Relatives	68	17.8
**Relationship with the surviving parent**		
Unchanged	202	52.8
Improved	121	31.6
Worsened	59	15.4

### Data Collection Tools

The sociodemographic data was collected using the Personal Information Form while the CTQ was used to collect data regarding the childhood trauma. The participants completed the data collections tools in 20 min on average. The data were collected in a four-month period (March-June) in 2015.

### Personal Information Form

The researchers developed the Personal Information Form based on the literature. Our choice of variables depended on our classification of variables related to the timing of parental death and variables that may be influenced by trauma. In this context, we asked socio-demographic questions like gender and age, which are intersecting categories both in the time of the parental death and current situation of participants. In addition, we inquired about the participants’ educational levels, marital status, and income, recognizing that their relationships may be explained by various variables, whether related to childhood trauma or not. To gain a deeper understanding of childhood trauma experiences, we incorporated variables related to the timing of parental death, such as the gender and age of the deceased parent, the child’s age at the time of parental death, the number of siblings, the caregiver after parental death, and the relationship with the surviving parent.

### Childhood Trauma Questionnaire (CTQ)

The Childhood Trauma Questionnaire (CTQ), which was developed by Bernstein et al. (1994) is used to investigate childhood traumas. The CTQ is an easy-to-apply measurement tool with proven validity and reliability which relies on retrospective self-report and which is useful in quantitatively evaluating individuals’ abuse and neglect. The reliability and validity analysis of the Turkish version of the CTQ, which has 28 items, was conducted by Şar et al. (2012). The scale has five sub-scales for sexual, physical and emotional abuse and emotional and physical neglect in childhood and the total score is calculated by combining the sub-scores. While the Cronbach’s alpha coefficient of the Turkish version is 0.93 and the test-retest correlation is 0.90, *p* < .001, the Cronbach’s alpha was found to be 0.761 in this study.

## Results

The data of the research was not normally distributed; thus, the Mann-Whitney U test was used for paired comparisons while the Kruskal-Wallis test was used to compare more than two groups. The data was found to be skewed to the right. Minumum, maximum and Cronbach Alpha values of CTQ and sub scores are given in Table 2.

**Table 2:** Minimum, maximum and cronbach alpha values of ctq and sub scores

	Mean	Standard Deviation	Maximum	Minimum	Cronbach Alpha
CTQ Total	40.45	4.73	59.20	27.60	0.761
Emotional Abuse	7.33	3.28	25.00	5	0.774
Physical Abuse	5.77	2.23	22.00	5	0.828
Physical Neglect	8.10	3.27	21.00	5	0.664
Emotional Neglect	10.91	4.64	25.00	5	0.827
Sexual Abuse	5.25	0.91	11.00	5	0.781

The results of the Mann-Whitney U test for the subscales of the CTQ are presented in Table 3. In the physical neglect subscale, gender was found to be associated with physical neglect, and the results showed that men were exposed to significantly more physical neglect compared to women (U = 13437,500, *p* < .05). As for marital status, it was found that the scores of physical neglect subscale differed significantly (*U* = 15672,000, *p* < .05), and the individuals who were physically neglected in childhood were currently married (*U* = 15672,000, *p* < .05). For the caregiver after parental death variable, a significant difference between physical and emotional neglect subscales was found and the individuals who were taken care of by their relatives were exposed to significantly more emotional abuse, physical and emotional neglect as compared to individuals who were taken care by the surviving parent (*p* < .05). For the deceased parent (mother or father) variable, a significant difference was found between emotional abuse, physical abuse, physical neglect, and emotional neglect sub-scales (*p* < .05). Accordingly, the individuals who lost their mother during childhood were significantly more abused and neglected. The age at the time of parental death was also a found to be significant in physical abuse (*U* = 1992, 500, *p* < .05), and the individuals who lost their parents at ages between 1 and 10 were exposed to significantly more physical abuse than those who lost their parents at ages between 11 and 18. The relationship between the variables presented in Table 3 and the sexual abuse subscale and the total score was not significant (*p* > .05).

**Table 3:** Results of the mann-whitney U test according to the subscales of the CTQ

CTQ	Gender	U	*P*	Marital Status	U	*P*	Caregiver After the Parental Death	U	*P*	Deceased Parents**	U	*P*	Age at the Time of Parental Death	U	*P*
Emotional Abuse	Female (*n* = 241)	16357.50	0.523	Single (*n* = 191)	17750.50	0.633	Other parent (*n* = 314)	8046.50	0.001^*^	Mother (*n* = 122)	4542.50	0.000^*^	Ages 1 and 10 (*n* = 132)	1967.50	0.055
	Male (*n* = 141)			Married (*n* = 191)			Relatives (*n* = 68)			Father (*n* = 243)			Ages 11 and 18 (*n* = 241)		
Physical Abuse	Female	16367.00	0.408	Single	18191.50	0.950	Other parent	9521.00	0.053	Mother	5420.50	0.019^*^	Ages 1 and 10	1992.50	0.029*
	Male			Married			Relatives			Father			Ages 11 and 18		
Physical Neglect	Female	13437.50	0.001^*^	Single	15672.00	0.016^*^	Other parent	8053.50	0.001*	Mother	4644.50	0.001^*^	Ages 1 and 10	2059.00	0.133
	Male			Married			Relatives			Father			Ages 11 and 18		
Emotional Neglect	Female	16170.50	0.430	Single	17416.50	0.444	Other parent	9027.00	0.045*	Mother	4599.50	0.001^*^	Ages 1 and 10	2073.50	0.150
	Male			Married			Relatives			Father			Ages 11 and 18		
Sexual Abuse	Female	16891.50	0.856	Single	17708.00	0.348	Other parent	10117.0	0.197	Mother	5861.00	0.185	Ages 1 and 10	2377.00	0.730
	Male			Married			Relatives			Father			Ages 11 and 18		
Total	Female	5840.00	0.098	Single	6662.50	0.270	Other parent	3018.00	0.081	Mother	424.00	0.000^*^	Ages 1 and 10	5562.00	.047*
	Male			Married			Relatives			Father			Ages 11 and 18		

** 122 participants lost their mothers, 243 participants lost their fathers and 17 participants lost their both parents. In order to investigate the impact of the loss of the mother and the loss of the father separately, 17 participants, who lost their both parents, were excluded from the data

The results of the Kruskal-Wallis test concerning the subscales of the CTQ are presented in Table 4. When the educational status is considered, it was found that there was a significant difference between physical and emotional neglect and abuse scores (*p* < .05). Accordingly, the primary-school graduates were significantly more neglected and abused compared to high-school and college graduates. In other words, the individuals who experienced emotional and physical abuse and neglect were not able to maintain their academic achievements. Moreover, the number of siblings was found to be significant in physical and emotional neglect (*p* < .05) as the individuals with more siblings experienced significantly more emotional and physical neglect compared to individuals with fewer siblings. Furthermore, the relationship with surviving parent was found to be significant in physical and emotional abuse and physical and emotional neglect (*p* < .05). Accordingly, the individuals whose relationship worsened with their surviving parents experienced significantly more emotional and physical abuse and emotional and physical neglect. While a significant relationship was found between the relationship with the surviving parent, the number of siblings, education and mean total scores (*p* < .05), there was no significant relationship between sexual abuse and the variables presented in Table 4 (*p* > .05).

**Table 4:** Results of the Kruskal-Wallis test according to the subscales of the CTQ

CTQ	Educational Status	X^2^	*P*	Relationship with the surviving parent	X^2^	*P*	Number of Siblings	X^2^	*P*
Emotional Abuse	**a.** Primary School (*n* = 149)	12.482	0.002^*^ ***a > b*** ***b > c***	**a.** Not changed (*n* = 202)	32.589	0.000^*^ ***c > b*** ***b > a***	**a.**1–3 (*n* = 163)	3.288	0.193
	**b.** High School (*n* = 155)			**b.** Improved (*n* = 121)			**b.**4–6 (*n* = 165)		
	**c.**College (*n* = 77)			**c.**Worsened (*n* = 159)			**c.**7+ (*n* = 51)		
Physical Abuse	**a.** Primary School	15.349	0.013^*^ ***a > b*** ***b > c***	**a.** Not changed	12.123	0.002^*^ ***c > b*** ***b > a***	**a.**1–3	1.337	0.512
	**b.** High School			**b.** Improved			**b.**4–6		
	**c.** College			**c.** Worsened			**c.**7+		
Physical Neglect	**a.** Primary School	28.003	0.00^*^ ***a > b*** ***b > c***	**a.** Not changed	26.034	0.000^*^ ***c > b*** ***b > a***	**a.**1–3	15.159	0.001^*^ ***c > b*** ***b > a***
	**b.** High School			**b.** Improved			**b.**4–6		
	**c.** College			**c.** Worsened			**c.**7+		
Emotional Neglect	**a.** Primary School	11.139	0.003^*^ ***a > b*** ***b > c***	**a.** Not changed	32.326	0.000^*^ ***a > b*** ***c > b***	**a.**1–3	12.347	0.002^*^ ***c > b*** ***b > a***
	**b.** High School			**b.** Improved			**b.**4–6		
	**c.** College			**c.** Worsened			**c.**7+		
Sexual Abuse	**a.** Primary School	0.246	0.889	**a.** Not changed	5.329	0.070	**a.**1–3	3.563	0.168
	**b.** High School			**b.** Improved			**b.**4–6		
	**c.** College			**c.** Worsened			**c.**7+		
Total	**a.** Primary School	10.604	0.005* **a > c** **c > b**	**a.** Not changed	20.110	***0.000*** ***c > b*** ***b > a***	**a.**1–3	10.476	0.005^*^ ***c > b*** ***b > a***
	**b.** High School			**b.** Improved			**b.**4–6		
	**c.** College			**c.** Worsened			**c.**7+		

## Discussion

This study aimed to investigate childhood traumas of adults who lost their parents in childhood or adolescence and to evaluate whether there is a relationship between age, marital status, educational status, family income, number of siblings, order of siblings, the deceased parent, the caregiver after the death of the parent, the relationship with the surviving parent, participant’s age at the time of parental death and childhood traumas. Before delving into the discussion of our results in the context of existing literature, it is important to acknowledge the potential presence of ecological fallacies. This arises from our association of variables such as education and marital status with childhood trauma, even though they may have correlations with other factors such as social status or variables unrelated to childhood trauma.

Having this limitation in our mind, we examined our results by categorizing variables into two groups: those related to the time after parental death experience, such as marital status, education, and gender, and those associated with the time of parental death, such as the number of siblings and the gender of the deceased parent. As it was mentioned previously, gender and age are intersecting categories in our results. The results showed that there is a significant relationship between gender, marital status, educational status, the caregiver after the parental death, the number of siblings, the deceased parent, the relationship with the surviving parent, the age at parental death, and childhood trauma of the participants. When analyzing the total CTQ score, it becomes evident that our participants can be classified as adults with invisible, unresolved trauma, as their CTQ score, which should fall between 25 and 125, averaged 40.45.

With respect to the time of the parental death, we found that there is a significant relationship between the age at the time of parental death and physical abuse. We found that the individuals who lost their parents at ages between 1 and 10 were exposed to significantly more physical abuse compared to those who lost their parents between ages of 11 and 18. As the literature suggests, children’s age significantly influences their perception and responses to death, and these reactions can vary depending on the child’s developmental stage. Consequently, it is argued that a child’s age at the time of parental death can impact their future psychopathology (Downdey, 2000). The literature further indicated that parental death is harder for younger children who depend on others for their care (Bildik, 2013). There are also studies indicating that there is no relationship between the child’s age at the time of parental death (*M*_*age*_ = 13) and their depression level (Uluğ, 2008). Considering the varied research findings, our results highlight the importance of social workers being cautious about identifying physical abuse in children aged 0–10. Children in this age group may be reluctant to disclose abuse due to concerns such as fear of abandonment and stigmatization. Social workers should also be careful about children between 10 and 18 since they are in a developmental and transition period in which risk of suicide increases and fewer coping mechnaisms are developed towards traumatic events (Hua et al., 2019). Here, the role of the caregiver after parental death becomes crucial, as the risk of abuse is associated with the caregiver for children who are not under protective services.

Regarding caregivers, we identified four related aspects: the gender of the deceased parent, the relationship with the surviving parent, caregiving by relatives, and the number of siblings. The literature highlights gender differences for both children and the deceased parent. It suggests that the death of fathers has more negative effects on sons, while the death of mothers creates more negative effects for daughters (Umberson, 2003; Marks, Jun, & Song, 2007). In relation to the children’s reactions to parental death, it is seen that maternal loss decreases daughters’ life satisfaction more than sons (Leopold & Lechner, 2015). Our results point out that the individuals who lost their mothers in childhood were exposed to more abuse and neglect compared to those who lost their fathers in childhood. Furthermore, we found that the individuals whose relationship with their surviving parent deteriorated experienced more physical and emotional neglect compared to other groups. Given that children typically direct their trust and attachment primarily toward their parents (Bildik, 2013), it is essential for the caregiving of the surviving parent to be a stabilizing factor, ensuring that the child does not lose this vital source of trust after the loss of one parent. In this context, research showed that positive emotional adjustment of the surviving parent leads to the positive adjustment of the child (Hope & Hodge, 2006) and the role of the surviving parent is a strong predictor of better general functioning in adulthood (Karydi, 2018).

Due to the eligibility criteria of our sample, the participants were taken care by either their surviving parent or their relatives. In terms of relatives, our result showed that the participants who were taken care of by their relatives after the parental death were exposed to significantly more emotional abuse and physical and emotional neglect. In the literature, several studies indicate that orphanhood and mistreatment are significant factors leading to leaving one’s home (Olsson et al., 2017). There are also studies indicating that positive communication between caregiver and the child serves as a predictor of the child’s psychological well-being (Wardecker et al., 2017). Here, it must be noted that in so-called traditional countries like Turkey, traditional family ties are still present, and caregiving is culturally seen as the responsibility of relatives or extended family when both parents pass away. Family centered care is further reinforced by the social work system, offering financial and material assistance. Although it is known that adults’ care of children decreases the risk of long-term psychological problems (Turkish Association of Psychologists, 2014), social workers should be careful about the fact that child’s own relatives can be the source of abuse and neglect, which can remain invisible as a secret of the family.

The number of siblings is another factor affecting the relationship between the child and the caregiver. We found a significant relationship between the number of siblings and physical and emotional neglect as well as the total score. This result can be explained either by the conscious mistreatment of the adults or the burden of stress on caregivers’ shoulders. Although parental death is seen as a stresful experience for children, it is also stresful for the caregiver. Just like the child, the caregiver experiences the loss of a loved one, faces an increase in domestic responsibilities, a decrease in the family budget, and a need for social, economic, and psychological support. Consequently, the caregiver may have difficulty finding enough time for the child, offering reduced support and affection, displaying impatience, and responding negatively to the child (Wolchick et al., 2006). Having more siblings under the responsibility of a caregiver may increase these potential behaviours and stress factors with which we can explain our results indicating that the individuals with more siblings were found to be significantly exposed to more physical and emotional neglect compared to those with fewer siblings. Based on this, we suggest that social workers should be aware that the higher number of children may create more pressure on caregivers, and social workers should facilitate programs not only related with children but also with caregivers. Furthermore, to change the perception of more children as burdens, they can work with the family system aiming to strengthen sibling ties and to create a perception with which having a sibling is seen as a source of support by other siblings and by family (Kalmjin & Leopold, 2018).

With respect to our result related to the relationship between the caregiver and the child, social workers’ trauma-informed practice should carefully consider the gender of the deceased parents, the relationship with the surviving parent and relatives, the risk of abuse and neglect, and the number of siblings when working with children and adults. We recommend the development of a follow-up system within social work centers, hospitals, school social work programs, or any social work system that assesses the relationship between children and caregivers after the loss of a parent. As a result, we believe that trauma-informed social work practice should meticulously account for factors such as the gender of the deceased parent, the relationship with the surviving parent, and the risk of abuse and neglect when working with bereaved children and the invisibly bereaved adults. Furhermore, social workers should design their intervention strategies to strengthen the bond between caregivers and children, along with interventions aimed at providing support to both parties.

Related to the current situation of the adult, we found a significant relationship between the adult’s gender, marital status, the level of education, and childhood trauma. In terms of gender, which is an intersecting variable during and after the traumatic event, we found that the men were exposed to significantly more physical neglect compared to the women. In the literature, there are different findings regarding the relationship between childhood trauma and gender. Some studies showed that the men were exposed to significantly more emotional and sexual abuse compared to women (Aslan & Alparslan, 1999; Çeçen, Eroğul, & Türk, 2013; Zeren et al., 2012). There are also studies indicating that physical abuse occurred equally among girls and boys, but in cases of sexual abuse, the majority were female (Koç et al., 2012). In another study on adults with the experience of institutionalization, gender makes a significant difference in the “emotional neglect” subscale of the CTQ, and it was observed that the women were neglected emotionally more than men (Kesen et al., 2016). Similarly, Tucci et al. (2010) found that the CTQ scores and the intensity of emotional, physical, and sexual abuse are more in women than men among drug and alcohol dependent patients. Yet, Güloğlu, Karaırmak and Emiral (2016) found that men were exposed to much more physical and emotional abuse and neglect as well as sexual abuse than women among a sample of university students. As seen, the literature suggests different results with different samples; therefore, we suggest that contrary to the general belief that girls are more open to abuse than boys, we suggest that social workers should consider all forms of neglect and abuse regardless of child’s gender while working with a child who experienced parental loss and with an adult who experienced parental loss in childhood or adolescence.

In terms of marital status, our results show that married participants were exposed to physical neglect in childhood significantly more than single participants. Different findings were reported on the relationship between marital status and childhood trauma in the literature. In a previous study conducted on the childhood trauma of college students, no significant relationship was found between marital status and physical, emotional and sexual abuse (Bostancı et al., 2006). Kesen et al. (2016) investigated individuals with experience of institutionalization and found that marital status was significantly related with emotional and physical abuse, physical neglect subscales, and the total score of participants. Beegle and Krutikova (2008) found that parental death leads girls to marry at younger ages than men. By highlighting different result of different studies and considering that different aspects of marriage decision can be affected by different factors like educational and socio-economic status, we suggest that physical neglect should be considered by social workers especially while working with children who want to marry at an early age and adults in family counseling.

When assessing neglect and abuse scores in relation to educational status, we observed significant differences in scores for physical abuse, neglect, and emotional neglect based on educational status. As our results indicate, the individuals who were abused and neglected in childhood were not able to continue their education or their education success is low. Levine, Gertler and Ames (2004) found that recent death of a parent increases school dropout possibility, which is two times more than children whose parents are alive. Feigelmen et al. (2017) emphasized that parental loss heightens the risk of suspension and expulsion, further noting that adolescents who have experienced parental bereavement often perceive their teachers as unsupportive, leading to reduced levels of hope regarding college attendance. Similarly, Berg et al. (2014) associated parental death with lower grades and school failure, and Gertler et al. (2004) highlighted the fact that parental loss and bereavement may lead to some difficulties for children about concentration on school work. When we analyze our findings in the context of this literature, it leads us to the conclusion that the trauma resulting from parental loss, combined with the lack of attention from caregivers and instances of neglect and abuse, may help explain the participants’ academic underachievement.

In conclusion, regarding education as well as the other variables, we suggest school social work, which is a project and not a part of social work system in Turkey, should be integrated into Turkish educational system with which educational and other needs of children and family can be followed up by social workers. In addition, school social workers can conduct awareness-raising activities in schools to educate both students and staff about the impact of parental loss on children, as well as the risk factors associated with abuse and neglect. Furthermore, social workers can facilitate peer support among children to enable bereaved children to develop coping mechanisms. Finally, we want to highlight that any social worker, regardless of whether they are a school social worker or not, should be aware of the effects of childhood traumas in adulthood and the factors related to these traumas. In line with this, we recommend that social workers integrate trauma-informed knowledge and practices into their intervention strategies.

## Conclusion

This research focused specifically on the individuals who experienced parental death before the age of 18 and who were not placed under protection or did not receive psychological help. We investigated childhood traumas of 382 participants and the relationship between the variables related to the time of parents’ death and after parents’ death. We found that the individuals who lost their parents at ages between 0 and 10, those who lost their mothers, those who were taken care of by their relatives, those who had more siblings, those whose relationship with the surviving parent worsened, and the men who lost their parents were exposed to significantly more neglect and abuse in childhood and adolescence. Moreover, we discovered that childhood trauma can be regarded as a significant factor contributing to the disruption of the educational progress of individuals who have experienced abuse and neglect. We also found that married participants experienced significantly more physical neglect in childhood than their single counterparts. Furthermore, those who experienced physical neglect in childhood were currently married. Consistent with our findings, the literature suggests a significant relationship between marital status and emotional abuse, physical abuse, as well as total scores on the Childhood Trauma Questionnaire. Additionally, the literature indicates that parental death is linked to girls marrying at younger ages than men. Based on the literature and our findings, we recommend that social workers consider the potential link between clients’ marriage decisions and experiences of neglect and abuse in trauma-informed practices. However, it’s important to note that our study did not explore the relationship between participants’ marriage decision processes and their trauma. Therefore, we suggest that this aspect be investigated further in future studies.

Even though numerous studies investigated childhood traumas in clinical samples and in connection with children under protection, our study differs from them in terms of its sample. Our study did not derive its sample from clinics or social work institutions and investigated the childhood trauma of individuals who lost their parents in childhood or adolescence in Turkey from the perspective of social work.

Our study has drawn conclusions regarding the relationship between various factors and types of abuse and neglect. These findings should be taken into consideration by professionals, especially social workers, in their trauma-informed practices. First, we have demonstrated that social workers can make a significant impact by taking into account the factors that should be considered to minimize the effects of childhood trauma. In this context, based on our findings, we suggest that when children between the ages of 0 and 10 experience parental death, they should be paid more attention due to their young age and developmental period. In addition, increased sensitivity should be shown to the individuals with more siblings and to the individuals who lost their mothers. Furthermore, we demonstrated that the caregiving responsibility of parents is more influential than that of relatives, and keeping a healthy relationship with the surviving parent may decrease the effect of trauma. Therefore, we concluded that facilitating and enhancing a healthy relationship between child and caregiver as well as the provision of support to the caregiver should be supported by social workers.

Our results showed that the children, who lost their parents, should be supported in their academic life, and their traumas should be considered in their marriage decisions. Accordingly, we highlight the reasons why school social work is a significant need for the children who lost their parents in Turkey. Addressing the needs of children, families, and their relationships, enhancing children’s academic success and their grieving process, strengthening support mechanisms, and promoting the psychosocial well-being of children are some of the contributions that school social work can offer. School social workers can also engage in activities aimed at raising awareness among children and teachers within the school environment.

While we advocate for protective measures to prevent children from becoming adults with unresolved, invisible trauma experiences, we recommend the integration of trauma-informed social work practices into social work systems. Social workers should actively address childhood trauma and related issues in their professional practices.

Finally, we suggest that future studies explore the reasons behind the prevalence of marriage among abused children, delve into their decision-making processes regarding marriage, and examine the types of abuse they experience. In addition, we recommend that qualitative studies should be conducted to investigate childhood traumas, possible intervention studies, trauma-informed practices of social workers, and school social work.

## Electronic Supplementary Material

Below is the link to the electronic supplementary material.

Supplementary Material 1

## Declarations

### Limitations

The participants’ retrospective self-report is the major limitation of our study, as they might have forgotten some details in their experiences. Likewise, it is possible that they might not be willing to share their experiences. Moreover, our sample is reached by snowball sampling which therefore may not fully represent the population. Finally, marital and educational status of the participants could be affected by other variables related or unrelated to childhood trauma.
