# Supplementary material for: Investigation of Childhood Traumas of Individuals Who Experienced Parental Death in Childhood or Adolescence in Turkey
Source: J Child Adolesc Trauma. 2024 Mar 15;17(3):899–909. doi: 10.1007/s40653-024-00629-2 (PMC11413260; doi:10.1007/s40653-024-00629-2)
Supplement: Supplementary file 1 — Supplementary Material 1 [file 40653_2024_629_MOESM1_ESM.docx]

**Responses to Reviewers’ Comments on the Manuscript with ref. no:** **WCAT-D-22-00107 and entitled:**

**“Investigation of Childhood Traumas of Individuals Who Experienced Parental Death in Childhood or Adolescence in Turkey”**

First, we would like to thank editors and Reviewer 3 for their constructive and valuable comments, which we sincerely believe, increase the quality, and enrich the content of the manuscript to a greater extent. We give a detailed description for each point raised by the Reviewer 3 and do our best to response each comment satisfactorily.

**Dear Reviewer 3,**

We genuinely appreciate your detailed feedback. We have incorporated the suggested revisions in the abstract, discussion, and conclusion sections. We have diligently highlighted these changes for your reference. You can see your comments and our revisions below.

Thank you for dedicating your time to our manuscript.

Best regards,

The authors.

**COMMENTS OF THE REVİEWER 3**

**Abstract**

**Page 1, Line 35/36
Change "the findings are expected to contribute trauma-informed social work" to "the findings contribute to trauma-informed social work."**

We changed the sentence and highlighted it with yellow, please refer to page 1.

**Discussion**

**Page 13, Line 53/54-55/56
Please change the sentence "Those who were sexually abused were girls in majority" to make it clearer. Do you mean that the study cited found that the majority of the sample who had been sexually abused were female?**

We changed the sentence to make it clearer. The revised sentence is as follows: “There are also studies indicating that physical abuse occurred equally among girls and boys, but in cases of sexual abuse, the majority were female”. Please refer to page 13.

**Conclusion**

**Page 16, Line 21/22**

**I presume this should read "those who were taken care OF by their relatives".**

Thank you for your feedback. We made the necessary corrections.

**Page 16, 17, 18**

**There are three separate references about "marriage decisions", for example: "we also found that marriage was more common among individuals who suffered from neglect and abuse in childhood and adolescence, which is an important data for trauma-informed practice and should be investigated further in future studies". Could you expand on this? Why is this a concern?**

**Page 18, first line: I think you should change "we suggest that future studies may investigate the reasons of the prevalence of marriage among abused children and the kind of abuse of the relatives". a) due to my above comment, and b) what do you mean "the kind of abuse of the relatives"?**

Thank you very much for your feedback. We made this comment considering that our research results, along with the literature, demonstrate a significant relationship between physical abuse and marriage, suggesting that trauma might lead to early marriage. Our findings on marriage are highlighted in green, and literature and comments are marked in yellow. We had already referenced the literature before the revision; now we have summarized the relevant section, adding it as a paragraph to the conclusion section. Additionally, we clarified that we did not inquire about any questions related to marriage decisions, leaving room for investigation in future research. We hope our explanation is sufficient. Please refer to page 16 to see our revision as follows:

1. "We also found that married participants experienced significantly more physical neglect in childhood than their single counterparts. Furthermore, those who experienced physical neglect in childhood were currently married. Consistent with our findings, the literature suggests a significant relationship between marital status and emotional abuse, physical abuse, as well as total scores on the Childhood Trauma Questionnaire. Additionally, the literature indicates that parental death is linked to girls marrying at younger ages than men. Based on the literature and our findings, we recommend that social workers consider the potential link between clients' marriage decisions and experiences of neglect and abuse in trauma-informed practices. However, it's important to note that our study did not explore the relationship between participants' marriage decision processes and their trauma. Therefore, we suggest that this aspect can be investigated further in future studies."
2. We changed the sentence as follows: “we suggest that future studies explore the reasons behind the prevalence of marriage among abused children, delve into their decision-making processes regarding marriage, and examine the types of abuse they experienced.”

**Limitations**

**There needs to be more consideration here. For example, it is not just that participants "might have forgotten"; they might not be willing to share their experiences. And have you considered potential limitations of snowball sampling?**

We revised the limitation section as follows:

The participants’ retrospective self-report is the major limitation of our study, as they might have forgotten some details in their experiences. Likewise, it is possible that they might not be willing to share their experiences. Moreover, our sample is reached by snowball sampling which therefore may not fully represent the population. Finally, marital and educational status of the participants could be affected by other variables related or unrelated to childhood trauma.

**Thank you again for dedicating your time to our manuscript and your valuable comments.**

**Best regards,**

**The authors.**
